# Two rare cases of acute myeloid leukemia with t(8;16)(p11.2;p13.3) and 1q duplication: case presentation and literature review

**DOI:** 10.1186/s13039-020-00507-0

**Published:** 2020-08-25

**Authors:** Meng Liu, Yuan Ren, Xianfu Wang, Xianglan Lu, Ming Li, Young Mi Kim, Shibo Li, Lijun Zhang

**Affiliations:** 1 Department of Hematology, The First Hospital of China Medical University, 155 Nanjing North Street, Shenyang, 110000 Liaoning People’s Republic of China; 2 Department of Pediatrics, University of Oklahoma Health Sciences Center, Oklahoma City, OK USA; 3 Department of Neurology, The Second Hospital of Jilin University, Jilin, People’s Republic of China

**Keywords:** 1q duplication, Acute myeloid leukemia, t(8;16)(p11.2;p13.3), Prognostic factor

## Abstract

**Background:**

Acute myeloid leukemia (AML) is a complex hematological disease characterized by genetic and clinical heterogeneity. The identification and understanding of chromosomal abnormalities are important for the diagnosis and management of AML patients. Compared with recurrent chromosomal translocations in AML, t(8;16)(p11.2;p13.3) can be found in any age group but is very rare and typically associated with poor prognosis.

**Methods:**

Conventional cytogenetic studies were performed among 1,824 AML patients recorded in our oncology database over the last 20 years. Fluorescence in situ hybridization (FISH) was carried out to detect the translocation fusion. Array comparative genome hybridization (aCGH) was carried out to further characterize the duplication of chromosomes.

**Results:**

We identified three AML patients with t(8;16)(p11.2;p13.3) by chromosome analysis. Two of the three patients, who harbored an additional 1q duplication, were detected by FISH and aCGH. aCGH characterized a 46.7 Mb and 49.9 Mb gain in chromosome 1 at band q32.1q44 separately in these two patients. One patient achieved complete remission (CR) but relapsed 3 months later. The other patient never experienced CR and died 2 years after diagnosis.

**Conclusion:**

A 1q duplication was detected in two of three AML patients with t(8;16)(p11.2;p13.3), suggesting that 1q duplication can be a recurrent event in AML patients with t(8;16). In concert with the findings of previous studies on similar patients, our work suggests that 1q duplication may also be an unfavorable prognostic factor of the disease.

## Background

Acute myeloid leukemia (AML) is a common disease characterized by immature myeloid cell proliferation and bone marrow failure, which can be subdivided into 9–11 pathogenetically different subtypes [1]. Over the past two decades, the incidence has increased by 30% [2, 3]. Furthermore, AML has poor long-term survival with a high relapse rate [4]. Therefore, AML represents a substantial health problem that requires strict monitoring and innovative treatment strategies. The development of newer, effective treatment strategies is necessary for AML patients.

To date, the detection of cytogenetic abnormalities has been regarded as a critical prognostic tool for AML treatment [5]. Hence, it is urgently necessary to identify chromosomal rearrangements in AML patients and provide the whole spectrum of cytogenetic abnormalities for AML [6]. According to the World Health Organization classification system updated in 2008, AML with recurrent genetic abnormalities including t(8;21)(q22;q22), t(11q23)/*MLL*, t(15;17)(q24;q21), inv(16)(p13.1q22), and t(16;16)(p13.1;q22) has been identified [7, 8]. Nonrandom chromosomal abnormalities, such as deletions and translocations, have been detected in approximately 52% of all adult AML patients. Moreover, chromosomal abnormalities have been recognized as genetic events that can cause and promote this disease [9]. Certain cytogenetic abnormalities, including t(8;21)(q22;q22), t(15;17)(q24;q21) and inv(16)(p13.1;q22), are associated with longer remission and survival, while alterations of chromosomes 5, 7, 11q23 and complex karyotypes are associated with poor response to therapy and shorter overall survival [10]. Chromosomal translocations such as t(15;17)/*PML*-*RARA*, t(8;21)/*RUNX1*-*RUNX1T1*, inv(16)/*CBFB*-*MYH11* and t(11q23)/*MLL* are usually found in AML patients [11, 12]. However, AML with t(8;16)(p11.2;p13.3)/*KAT6A*-*CREBBP* is a very rare AML subtype and can be found in any age group, from infancy to the eighth decade of life, with a female predominance [13–17]. A majority of adult patients with t(8;16)(p11.2;p13.3) are therapy related [14–17], and pediatric patients tend to be de novo [13]. There are approximately 160 cases reported in the literature [13–17], and the first t(8;16)(p11.2;p13.3) in an infant was described in 1983 [18]. Some AML patients with t(8;16) (p11.2;p13.3) have a bleeding tendency and disseminated intravascular coagulopathy, which are overlapping clinical features that mimic acute promyelocytic leukemia (APL) [17]. Unlike APL, AML with t(8;16)(p11.2;p13.3) has an unfavorable treatment response and outcome [14, 19]. As a sole chromosomal anomaly, t(8;16)(p11.2;p13.3) is found in more than 50% of reported cases, and one or more additional chromosomal anomalies can be seen in the remaining cases [20]. The most common secondary chromosomal anomalies are total or partial trisomy 8 and monosomy 7 or deletion of the long or short arm of chromosome 7 [13–16, 19]. Comparatively, the gain of 1q in variable sizes has also been frequently noticed in patients with t(8;16)(p11.2;p13.3) in these large studies [13–15, 19].

Recurrent cytogenetic abnormality t(8;16)(p11.2;p13.3) is seldom associated with AML, and the 1q duplication in AML patients with t(8;16)(p11.2;p13.3) has never been discussed. In the present study, a total of 1,824 de novo or treatment-related AML patients were collected from our laboratory oncology database. Among them, three patients were detected with t(8;16)(p11.2;p13.3)/*KAT6A*-*CREBBP*, and two of these three showed an additional copy of partial chromosome 1q.

## Methods

### Patients

This study was approved by the Institutional Review Board (IRB) of Oklahoma University (IRB Number: 2250). A total of 1,824 AML patient samples were studied cytogenetically from 2000 to 2019 at the Genetics Laboratory of Oklahoma University Health Sciences Center. Bone marrow samples were obtained from three of the 1,824 patients who had t(8;16)(p11.2;p13.3).

### Conventional cytogenetic analysis

Short-term cultures of unstimulated bone marrow samples were established and harvested according to standard laboratory protocols. Karyotype analysis was performed using Giemsa and trypsin techniques for G-banding. The cytogenetic abnormalities were described according to the International System for Human Cytogenetic Nomenclature (ISCN 2016).

### Fluorescence in situ hybridization analysis

Fluorescence in situ hybridization (FISH) assays were performed according to the manufacturer’s instructions in combination with our established laboratory protocols. A *PML/RARA* dual-color, dual-fusion translocation probe (Abbott Molecular Inc., Des Plaines, IL, USA), subtelomere-specific probes for chromosome 3 p-arm and q-arm, and whole chromosome painting (WCP) probes for chromosomes 1, 3 and 14 were purchased from Cytocell Ltd, NY, USA. A spectrum green-labeled probe mapping to the 8p11.21 region and a spectrum orange-labeled probe mapping to the 16p13.3 region were created in house with the following BAC/PAC clones: RP11-642I24[chr8: 41,676,336-41,856,494(hg19)] and RP11-589C21[chr8: 41,873,702-42,036,222(hg19)], RP11-619A23[chr16: 3,720,076-3,914,571(hg19)] and RP11-95J11[chr16: 3,860,374-4,025,510(hg19)] (Children’s Hospital Oakland Research Institute, Oakland, CA, USA). The *KAT6A* gene located on 8p11.21 and the *CREBBP* gene located on 16p13.3 were covered by the green-labeled and red-labeled home-brewed probes, respectively. All probes were validated before use. Chromosome spreads were counterstained with 4,6-diamidino-2-phenylindole (DAPI4) in antifade medium (Vector Laboratories Inc., CA, USA). Digital images carrying specific hybridization signals were captured and processed on CytoVision version 7.0 (Applied Spectral Imaging, Carlsbad, CA, USA).

### aCGH analysis

Genomic DNA was extracted from each of the three patients’ bone marrow pellets according to the standard operating procedure using the phenol and chloroform method with a commercially available DNA extraction kit (Puregene blood kit, Qiagen, Valencia, CA) or Nucleic Acid Isolation System (QuickGene-610L, FUJIFILM Corporation, Tokyo, Japan). Two aCGH platforms, NimbleGen and Agilent, were used in this study. For the NimbleGen aCGH platform, human reference genomic DNA was purchased from Promega Corporation (Promega Corporation, Madison, WI, USA). The patient’s DNA and the reference DNA were labeled with either Cyanine 3 (Cy-3) or Cyanine 5 (Cy-5) by random priming, and then equal quantities of both labeled products were mixed and loaded onto a 720 K oligonucleotide chip (Roche NimbleGen Inc., Madison, WI, USA) to hybridize at 42 °C for 40 h in a MAUI hybridization system (BioMicro Systems, Salt Lake City, UT) according to the manufacturer’s protocols with minor modifications. The slides were washed with washing buffers (Roche NimbleGen Inc.) after hybridization and scanned using a Roche Scanner MS 200 Microarray Scanner (Roche NimbleGen Inc.). Images were analyzed using NimbleScan software version 2.6 and SignalMap software version 1.9 (Roche NimbleGen Inc.). The genomic positions were determined using GRCh36/hg18, UCSC Genome Browser. For the Agilent aCGH platform, human reference genomic DNA was purchased from Agilent Corporation (Agilent Corporation, Santa Clara, CA, USA). The patient’s DNA and the purchased reference DNA were labeled with either Cyanine 3 (Cy-3) or Cyanine 5 (Cy-5) by random priming (Agilent Corporation). Patient DNA (labeled with Cy-3) was combined with a normal control DNA sample (labeled with Cy-5) of the same sex and hybridized to an Agilent 2 × 400 K oligo microarray chip (Agilent Technologies) by incubating in an Agilent Microarray Hybridization Oven (Agilent Technologies). After 40 h of hybridization at 67 °C, the slides were washed and scanned using the NimbleGen MS 200 Microarray Scanner (Roche NimbleGen Inc.). Agilent’s CytoGenomics 2.7 software (Agilent Technologies.) was applied for data analysis. The genomic positions were determined using GRCh37/hg19, UCSC Genome Browser.

### Case presentation

*Case 1* An 82-year-old male presented with anemia was referred to us for AML evaluation. His subsequent lab results and hospital records were not available in our clinical database.

*Case 2* A 28-year-old female presented with disseminated intravascular coagulopathy was referred to rule out APL. Her complete blood examination and bone marrow aspirate smears were not available. Flow cytometry revealed 57% monocytic cells positive for CD4, CD11b (partial), CD13 (bright), CD14 (partial), CD15, CD33 (bright) and HLA-DR (partial) but negative for CD3, CD7, CD34, CD117, MPO and TdT, consistent with a diagnosis of AML with monocytic differentiation (subtype M5). The patient achieved hematological CR on day 15 and cytogenetic CR on day 33 after induction chemotherapy and then relapsed 3 months later.

*Case 3* A 69-year-old female with a medical history of breast cancer after lumpectomy, chemotherapy, and radiation presenting with generalized weakness, pancytopenia, and fever was referred to us for disease progression evaluation. A complete blood examination showed a white blood cell count of 216 × 10^9^/L with 53% blasts, a hemoglobin count of 66 g/L and a platelet count of 31 × 10^9^/L. Her bone marrow aspirate smear demonstrated over 90% myeloblasts. Flow cytometry revealed that 69% of the blast cells expressed CD45 (moderate), CD34 (dim), CD38, HLA-DR, CD13, CD15, and CD33 and were negative for CD117, consistent with a diagnosis of AML with monocytic differentiation (subtype M5). The patient started consolidation chemotherapy but had spontaneous regression and died 2 years after AML diagnosis.

## Results

**Fig. 1:**
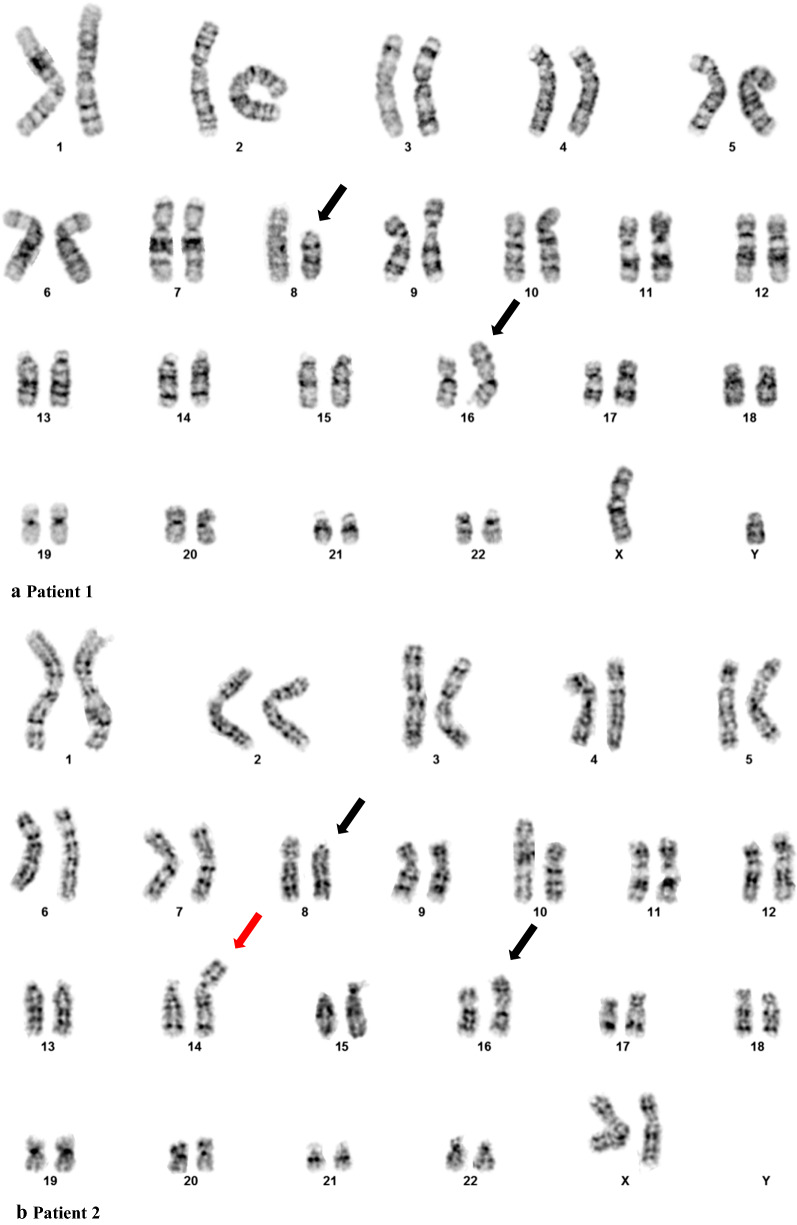

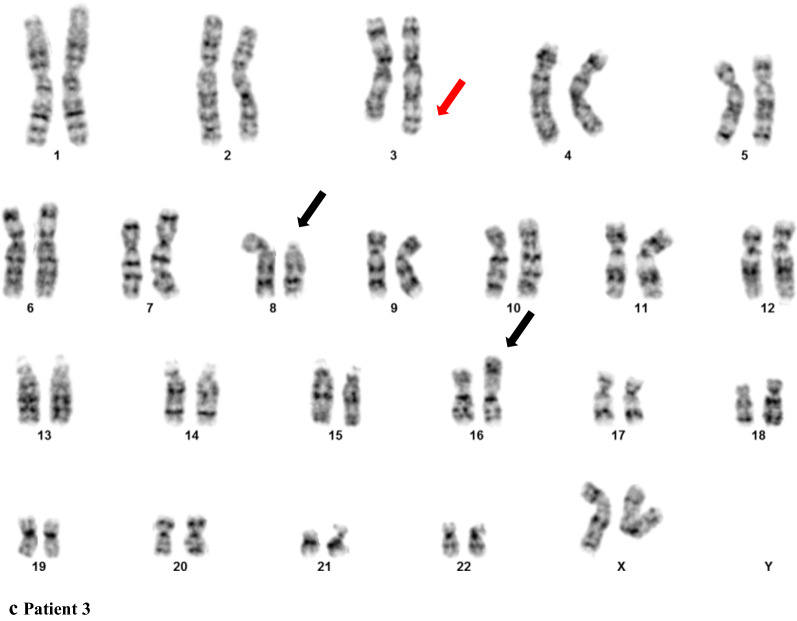
Representative abnormal karyotypes of three patients with t(8;16)(p11.2;p13.3). **a** Karyotype of patient 1 showing 46,XY,t(8;16)(p11.2;p13.3) as the sole abnormality; **b** and **c** Karyotypes of patients 2 and 3 showing 46,XX,t(8;16)(p11.2;p13.3) and an additional chromosome segment attached to the short arm of chromosome 14 and the long arm of chromosome 3, respectively. Translocated derivatives 8 and 16 are indicated by black arrows, and derivatives 14 and 3 are indicated by red arrows

In case 1, routine chromosome analysis detected an abnormal karyotype with a translocation between the short arms of chromosomes 8 and 16 (Fig. 1a) in 17 of 20 cells, consistent with a diagnosis of AML with t(8;16)(p11.2;p13.3). The nomenclature of the cytogenetic findings in patient 1 was t(8;16)(p11.2;p13.3)[17]/46,XY[3]. No other consistent karyotypic aberrations were detected. Thus, this male patient was excluded from subsequent FISH and aCGH analyses.

**Fig. 2:**
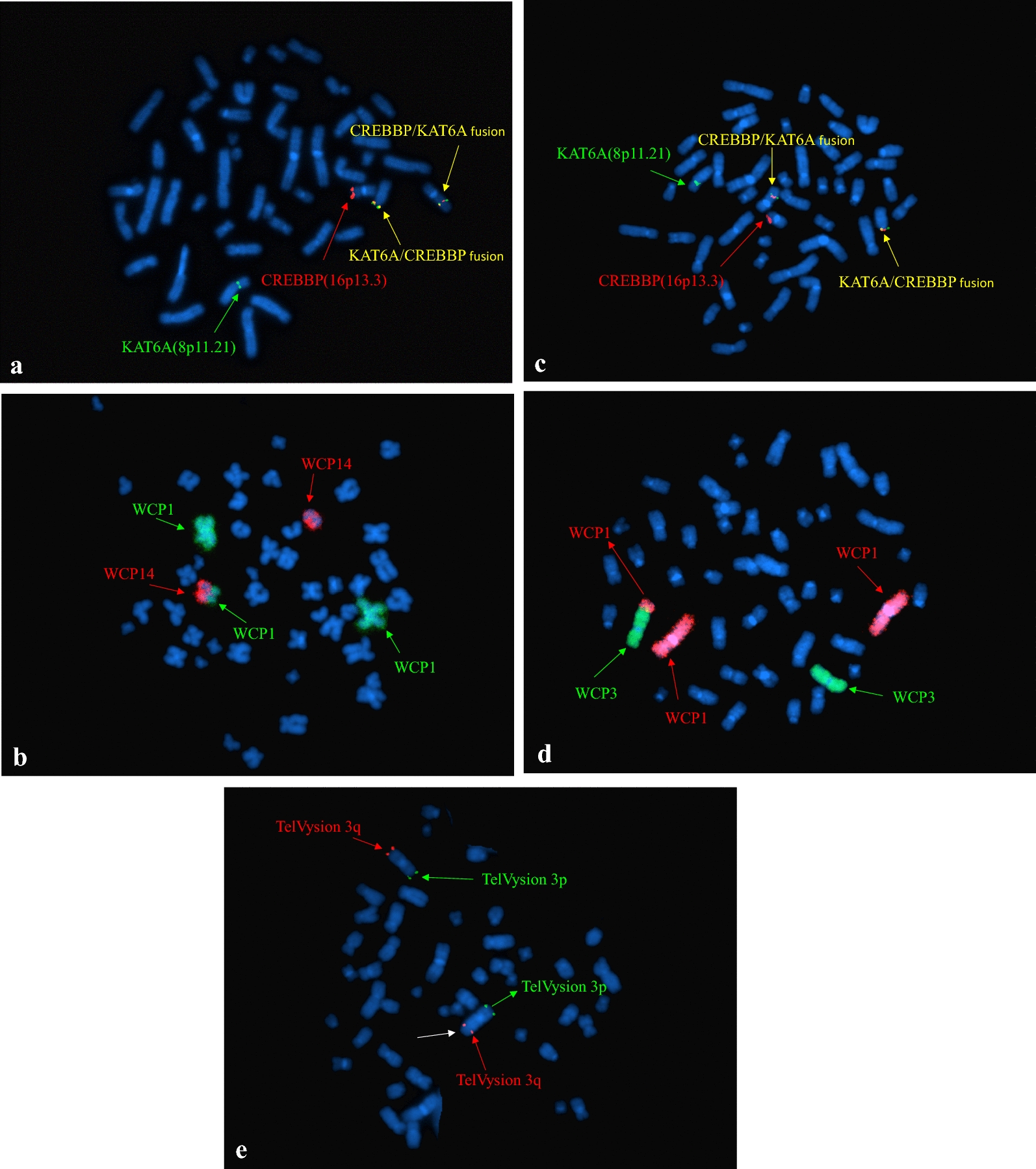
Metaphase FISH of patient 2 (**a**) and 3 (**c**) showing *KAT6A/CREBBP* fusion signals. WCP FISH indicating the extra chromosomal materials on chromosome 14 and chromosome 3 were both from chromosome 1 (**b** and **d**). No loss of the end portion of the chromosome 3 long arm was indicated (**e**)

**Fig. 3:**
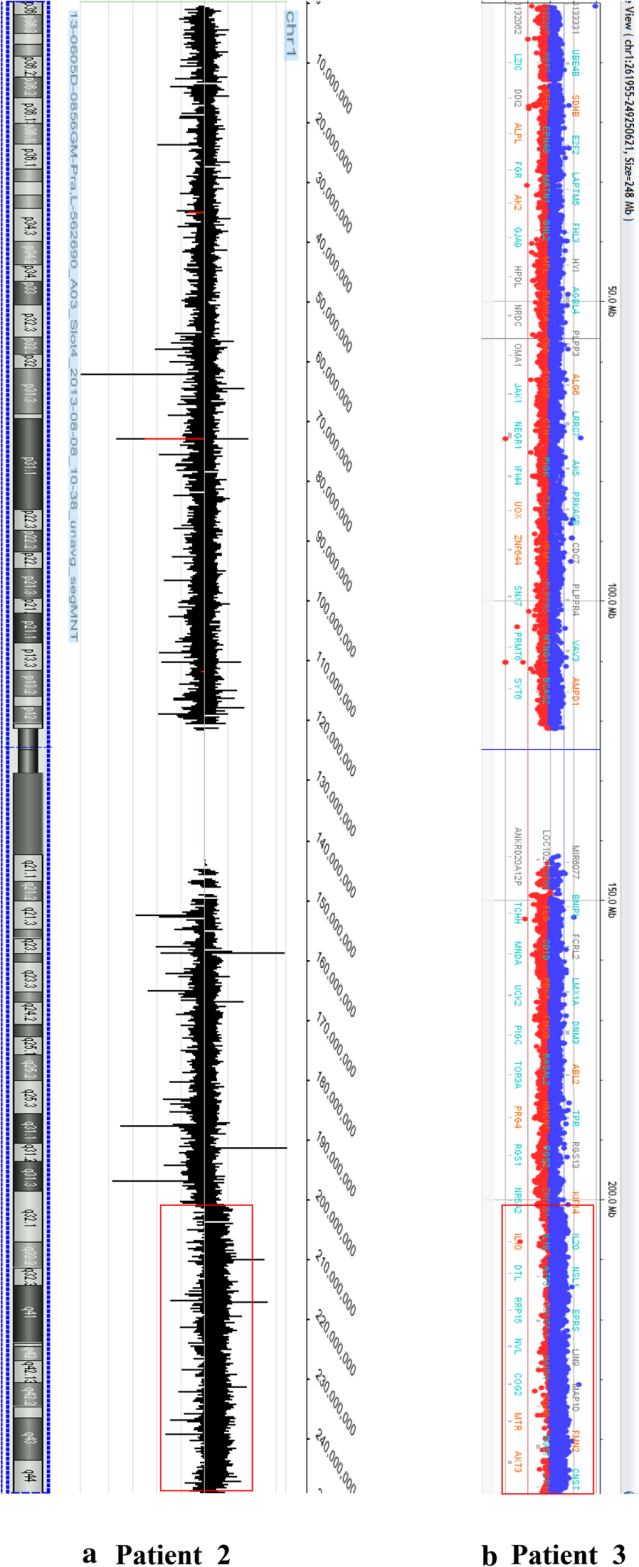
aCGH results of patient 2 and patient 3 showing partial 1q gain; duplicated 1q regions are indicated by red frames

In case 2, chromosome analysis demonstrated the same chromosome rearrangement between 8 and 16 in all 20 cells. Besides, 11 of these cells showed an extra chromosome segment attached to chromosome 14 (Fig. 1b). The karyotypes in patient 2 were described as 46,XX,t(8;16)(p11.2;p13.3), add(14)(p11.2)[11]/46,XY[9]. Negative FISH results for t(15;17)(q24;q21)/*PML*-*RARA* further ruled out a diagnosis of APL (data not shown). Metaphase FISH analysis confirmed the t(8;16)(p11.2;p13.3)/*KAT6A*-*CREBBP* fusion and demonstrated a part of chromosome 1 on chromosome 14 (Fig. 2a and b). In addition to characterizing the extrachromosomal 1 material, aCGH was carried out. aCGH confirmed the FISH findings and detected a 46.7 Mb gain from chromosome 1 at bands q32.1q44 (201,304,064-248,102,389 bp, GRCh36/hg18, USCS Genome Browser) (Fig. 3a).

In case 3, t(8;16)(p11.2;p13.3) with a gain of a similar chromosome segment on the long arm of chromosome 3 was detected in 18 of 20 cells by karyotyping analysis (Fig. 1c). FISH confirmed the *KAT6A*-*CREBBP* fusion and revealed additional chromosome 1 material (Fig. 2c and d). Loss of the end portion of the chromosome 3 long arm was not found by FISH (Fig. 3e). aCGH further detected a gain from chromosome 1 at bands 1q32.1q44 (201,408,592-251,323,872 bp, GRCh37/hg19, UCSC Genome Browser) (Fig. 3b). The molecular size was 49.9 Mb.

## Discussion

AML is one of the most common diseases characterized by the proliferation of blast cells in bone marrow or peripheral blood, which accounts for approximately 30% of adult leukemia cases. As reported previously, common chromosomal translocations such as t(8;21)/*RUNX1*-*RUNX1T1*, t(15;17)/*PML*-*RARA*, and inv(16)/*CBFB*-*MYH11* are frequently observed, and numerous uncommon chromosomal aberrations also exist in AML [12]. The detection of these fusion transcripts is important for the diagnosis and progression monitoring of AML patients [21].

In previous large studies, approximately 160 AML cases with t(8;16)(p11.2;p13.3) have been reported [13–20]. Among them, 9 cases showed a gain by 1q of variable sizes [13–15, 19]. As an uncommon entity, t(8;16) accounts for 0.2–0.4% of all cases of AML [13–20]. In our study, three patients with t(8;16)(p11.2;p13.3) were identified: one man and two women. The two women were both diagnosed with AML (subtype M5) and showed an extra copy of 1q at the same bands (q32.1q44), which were different from the nine reported cases above. The clinical features and cytogenetic data of the 11 cases of AML with t(8;16)(p11.2;p13.3) and 1q duplications are summarized in Table 1. To the best of our knowledge, this is the first study of the delineation of 1q duplication by aCGH in AML patients with t(8;16)(p11.2;p13.3).

**Table 1:** The previously reported AML cases with t(8;16)(p11.2;p13.3) and 1q duplication

	Sex	Age (years)	FAB type	Karyotype	1q Bands	Outcome (years)	Last state
Case 2	F	28	M5	46,XX,t(8;16)(p11.2;p13.3), add(14)(p11.2)[11]/46,XX[9]	1q32.1q44	CR after induction Relapsed 3 months later	Alive
Case 3	F	69	M5	46,XX,t(8;16)(p11.2;p13.3)[2]/46,idem,add(3)(q?27)[18]	1q32.1q44	spontaneous regression	Died
Haferlach et al.	F	39	M5a	45,XX,t(8;16)(p11;p13),der(10;13)(q10;q10)[10]/46,XX,der(7)t(1;7)(q21;q35),t(8;16)(p11;p13)[2]/46,XX[1]	1q21	NA	NA
Diab et al.	M	14.5	M4	46,XY,+1,del(1)(p22),t(8;16)(p11;p13),-10,der(14)t(10;14)(q11.2;p11.2)[8]/47,XY,del(1)(q11),+der(1)t(1;8)(p11;q11.2)x2,+i(5)(p10),-8,-10,der(14)t(10;14)(q11.2;p11.2),der(16)t(8;16)	Partial 1q gain	CR for 10.5	Dead
Diab et al.	F	14.2	M4/5	5,XX,t(8;16)(p11;p13),-18,der(21)t(1;21)(q12;p13)[4]/46,XX[16]	1q12	CR for 5	Alive
Diab et al.	F	1.2	M4	46,XX,t(8;16)(p11;p13)[3]/46,idem,der(10)t(1;10)(q11;p11)[5]/46,idem,add(7)(p21),der(10)t(1;10)(q11;p11)[2]/46,idem,add(7)(p21)[2]/46,XX[2}	1q11	CR for 0.6	Died
Diab et al.	F	14.1	M4	46,XY,t(8;16)(p11;p13),der(14)t(1;14)(q31;p11)[20]*	1q31	CR for 11.5	Alive
Diab et al.	F	7.3	M5	46,X,der(X)t(X;1)(q26;q23),t(8;16)(p11;p13),der(11)t(11;11)(p11;q13)	1q23	NA	NA
Xie et al.	M	28	M4	46,XY,der(3)t(3;8)(q27,q13),del(6)(p22),t(8;16)(p11.2;p13.3),del(10)(q21q25),add(13)(p11.2),del(16)(p12),del(20)(p11.2),del(20)(q11.2q13.3)[4]/46,idem,del(1)(p35p36.3),del(15)(q23),add(19)(p13.1)[2]/46,XY,t(8;16)(q27;q13),del(12)(q21q24.1),del(13)(q21q31),-16,der(19)t(1;19)(q32;p13.3),+mar[3]/46,XY,del(6)(p22),t(8;16)(p11.2;p13.3)[cp2]/46,XY[9]	1q32	CR for 7 months	Dead
Brown et al.	M	71	M4	47,X,der(Y)t(Y;1)(q12;q21), +6,t(8;16) (p11;p13)[6]/47,idem,del(13)(q3q3) [checked with CAD data]	1q21	No CR	Died 1 month after treatment
Brown et al.	F	1.2	M4	46,XX,t(8;16)(p11;p13)[3]/46,idem,der(10)t(1;10)(q11;p11)[5]/46,idem,add(7)(p21),der(10)t(1;10) (q11;p11) [2]/46,idem,add(7)(p21) [2]/46,XX [2]	1q11	Early remission after course 1. Relapsed at 5 months and 7 months after diagnosis	Died

*AML* acute myeloid leukemia, *FAB* French–American–Britishh, *M* male, *F* female, *NA* not available, *CR* complete remission

AML patients with this abnormality often show unique clinical and biological characteristics [22]. Compared with the current categories t(15;17), t(8;21), inv(16), and t(11q23) in AML, t(8;16) is clustered closer to t(11q23) and shares commonly expressed genes [15]. Xie et al. reported 15 adult AML cases with t(8;16)(p11.2;p13.3), indicating that t(8;16)(p11.2;p13.3) commonly exhibits monoblastic or myelomonocytic differentiation and arises in patients with a history of cytotoxic-treated cancer. Patients with de novo AML with t(8;16) or treatment-related AML with t(8;16) without adverse prognostic factors have a good outcome [14]. Identifying adverse prognostic factors is of importance to the choice of therapy and evaluation of survival in AML patients with t(8;16).

Over the past 15 years, cytogenetic and molecular technologies have largely promoted the efficiency of the identification and characterization of this disease [5]. Compared with conventional cytogenetic analysis and FISH methods, aCGH is an attractive method for the investigation of cancer genomes [23]. aCGH has higher resolution, simplicity, high reproducibility, shorter turnaround time and precise mapping of aberrations. Most importantly, it avoids the need for cell culture and dividing cells [24–26]. Furthermore, aCGH chromosomal analysis facilitates rapid detection and duplication of cytogenetic abnormalities previously undetectable by conventional cytogenetics [27]. In our investigation, we applied aCGH to characterize the additional chromosome 1 materials in patients 2 and 3 and interestingly found that the two patients revealed the same extra copy of 1q at bands q32.1q44. Patients with 1q duplication have also demonstrated a wide range of multiple malformations, such as intellectual disability, macrocephaly, large fontanels, prominent foreheads, broad flat nasal bridges, high-arched palates, retrognathia, low-set ears, and cardiac defects [28, 29]. More recent studies have shown that a 1q gain is also related to a portion of solid tumors. For instance, the gain of 1q is well known as a poor prognostic biomarker of Wilms tumor [30], and it plays an important role in predicting poor clinical outcome in patients with thyroid carcinoma as well [31]. In addition, patients with a 1q duplication showed worse survival and high risk in acute leukemia, Burkitt lymphoma, and myeloproliferative neoplasms [32–36]. The outcomes of 1q duplication in the nine reported AML patients with t(8;16)(p11.2;p13.3) are summarized in Table 1. Seven patients’ data were available. These seven patients (two adult and five pediatric) all received induction chemotherapy, and six achieved CR. At the time of last follow-up, two adult patients and three of five pediatric patients had died. Only two pediatric patients were alive. We reported two adult patients here: patient 2 achieved CR but relapsed 3 months later, and patient 3 had spontaneous regression and died 2 years after diagnosis. Taken together, the findings suggest that 1q duplication might be associated with adverse outcomes in AML patients with t(8;16)(p11.2;p13.3). However, the significance of the 1q duplication in AML with t(8;16) needs to be further investigated. Since such changes have been seldom reported, the pathogenic effects of 1q duplication in AML patients with t(8;16)(p11.2;p13.3) require more studies to be delineated.

## Conclusion

Three patients were detected with t(8;16)(p11.2;p13.3) from an 1,824 AML patient database. Two female patients were identified with a 1q duplication by FISH and aCGH analyses. Combining our investigation with the findings of published studies, we conclude that 1q duplication is a recurrent finding in AML patients with t(8;16). Our data also suggest that 1q duplication might be associated with unfavorable prognosis in these cases. The understanding of cytogenetic data would contribute to the diagnosis and treatment evaluation of AML.

## Data Availability

All data generated or analyzed during this study are included in this published article.

## Abbreviations

AML: Acute myeloid leukemia
aCGH: Array comparative genomic hybridization
FISH: Fluorescence in situ hybridization
APL: Acute promyelocytic leukemia
WCP: Whole chromosome painting
CR: Complete remission
